# Efficacy Endpoint Standardization in Adult Primary CNS Tumor Trials: Integrating Regulatory Science and Clinical Perspectives in the RANO 2.0 Era

**DOI:** 10.3390/cancers18121872

**Published:** 2026-06-08

**Authors:** Shinya Watanabe, Takahiro Nonaka, Masanobu Yamada, Makoto Maeda, Narushi Sugii, Yoshihiro Arakawa, Koichi Hashimoto, Eiichi Ishikawa

**Affiliations:** 1 Department of Neurosurgery, Mito Kyodo General Hospital, Tsukuba University Hospital Mito Area Medical Education Center, Mito 310-0015, Japan; 2 Institute of Medicine, University of Tsukuba, Tsukuba 305-8575, Japan; narushi-sugii@md.tsukuba.ac.jp (N.S.); e-ishikawa@md.tsukuba.ac.jp (E.I.); 3 Department of Clinical Research Governance, The University of Tokyo Hospital, Tokyo 113-0033, Japan; nonaka.takahiro@mail.u-tokyo.ac.jp; 4 Tsukuba Clinical Research and Development Organization, University of Tsukuba, Tsukuba 305-8576, Japan; yamada-masanobu@md.tsukuba.ac.jp (M.Y.); arakawa-tky@umin.ac.jp (Y.A.); koichi.hashimoto@md.tsukuba.ac.jp (K.H.); 5 Department of Pharmacy, National Cancer Center Hospital, Tokyo 104-0045, Japan; makmaeda@ncc.go.jp

**Keywords:** regulatory science, RANO 2.0, neuro-oncology, clinical trial, efficacy endpoints, glioblastoma, meningioma, CNS tumor, Phase II trial, objective response rate

## Abstract

**Background/Objectives:** Efficacy endpoint selection in adult primary central nervous system (CNS) tumor trials remains challenging because conventional solid tumor frameworks do not adequately capture the anatomical, radiographic, and biological complexity of brain tumors. In particular, postoperative irregular residual lesions, non-enhancing tumor components, and treatment-related imaging changes complicate the interpretation of objective response and progression. This narrative review examines the current landscape of endpoint selection in adult primary CNS tumor trials and discusses strategies for standardization in the Response Assessment in Neuro-Oncology (RANO) 2.0 era from integrated regulatory science and clinical perspectives. **Methods:** This study was conducted as a narrative review intended to provide a regulatory science-oriented synthesis of efficacy endpoint evaluation in adult primary CNS tumor trials. The literature search primarily utilized PubMed and ClinicalTrials.gov, supplemented by major consensus guidelines, pivotal clinical trials, and regulatory documents from the major regulatory authorities, including those in the United States, Europe, and Japan, published over the past two decades. Search terms included combinations of keywords such as “brain tumor,” “glioblastoma,” “meningioma,” “Phase I,” “Phase II,” “efficacy endpoint,” and “RANO”. In addition to the literature synthesis, this review incorporates findings from our previously published empirical analyses regarding endpoint selection in Phase II glioblastoma trials, Phase II meningioma trials, and Phase I brain tumor trials. **Results:** Response Evaluation Criteria in Solid Tumors-based response assessment remains fundamentally limited in neuro-oncology. Across recent early-phase trials, substantial heterogeneity persists in endpoint selection and in the operational definitions of objective response rate, progression-free survival, and progression. RANO 2.0 is intended to provide a more unified and implementation-oriented framework by refining baseline definition, progression confirmation, non-enhancing lesion interpretation, and the role of minor response, while improving compatibility with contemporary molecular classification and immunotherapy-era trial design. However, important implementation challenges remain, including potential reproducibility concerns in complex imaging assessments, operational complexity, imaging standardization, and the need for independent central review. **Conclusions:** Standardization of endpoint strategies in adult primary CNS tumor trials should move beyond simple adoption of conventional solid tumor metrics toward a disease-specific, harmonized framework integrating imaging, clinical context, tumor biology, and regulatory interpretability. This review provides a regulatory science-oriented synthesis linking response assessment criteria, early-phase adult primary CNS tumor trial endpoint trends, empirical analyses, and emerging endpoint frameworks. RANO 2.0 represents an important step toward this goal, but it should be regarded as an evolving framework that requires continued validation, international collaboration, and implementation-focused refinement.

## Simple Summary

Evaluating whether a treatment works is essential in developing new therapies for brain tumors. However, unlike tumors in other organs, brain tumors are difficult to assess using standard methods because they often show complex imaging patterns, including non-enhancing lesions, treatment-related changes, and temporary worsening that does not reflect true tumor growth. As a result, different clinical trials use different evaluation methods, making it difficult to compare results, thereby slowing drug development. This review explains the limitations of current assessment approaches and discusses the evolution of brain tumor–specific evaluation criteria, particularly the latest Response Assessment in Neuro-Oncology 2.0 framework. It also highlights the need for more consistent and disease-specific evaluation strategies that integrate imaging findings, clinical conditions, and treatment context. Improving the way treatment effects are measured may help accelerate the development of effective therapies and support better clinical and regulatory decision-making in brain tumor research.

## 1. Introduction

### 1.1. Unique Challenges in Efficacy Evaluation for CNS Tumors

Recent advances in oncology drug development—particularly the emergence of molecularly targeted agents directed at specific driver mutations—have substantially transformed clinical development strategies. In response to these global trends, the Japanese regulatory authority issued the “Guideline on Clinical Evaluation Methods for Anti-cancer Drugs” in 2021, outlining updated development strategies that reflect the evolving landscape of oncology drug development [1]. These agents can produce striking antitumor effects in rare molecular subtypes, where conducting conventional confirmatory trials is often infeasible.

In oncology clinical trials, the gold standard for efficacy evaluation remains hypothesis testing based on overall survival (OS) and progression-free survival (PFS) for Phase III trials [1] and objective response rate (ORR) based on RECIST (Response Evaluation Criteria in Solid Tumors) [2] for Phase II trials. However, especially in Phase II trials, there is a fundamental distinction between CNS (central nervous system) tumors and non-CNS solid tumors. In many non-CNS solid tumor settings, RECIST-based ORR has been widely used as an early efficacy endpoint in Phase II trials [3], although its surrogate value is not uniform across tumor types and may vary depending on tumor biology, treatment mechanism, and clinical context.

In contrast, for CNS tumors, no international consensus currently exists regarding the appropriateness of RECIST, and a variety of evaluation methods are currently in use, including response rate based on Response Assessment in Neuro-Oncology (RANO) criteria [4] and Immunotherapy Response Assessment in Neuro-Oncology (iRANO) criteria [5], as well as OS and PFS rates, indicating that regulatory harmonization has yet to be achieved. Even with recent efforts to improve reproducibility through automated imaging analysis, fundamental inconsistencies in evaluation methodology remain [6]. In Phase II trials for CNS tumors, especially glioblastoma (GBM)—a highly treatment-resistant tumor with extremely poor prognosis, as highlighted in previous reviews [7]—and malignant and/or recurrent meningioma—where therapeutic development has been relatively active among primary CNS tumors—several unique challenges hinder consistent efficacy assessment. These CNS tumor–specific issues [8,9,10] highlight the fundamental limitations of applying conventional solid tumor evaluation approaches to CNS tumors and underscore the need for a disease-specific framework for endpoint determination [11].

### 1.2. Historical Development of Brain Tumor-Specific Imaging Response Criteria

Objective measurement of tumor shrinkage has long been a cornerstone of efficacy assessment in anticancer drug development. This principle also applies to CNS tumors. One of the earliest formal efforts in this direction was the Macdonald criteria in 1990 [12], which defined tumor response based on two-dimensional measurements (maximum diameter × perpendicular diameter) of enhancing lesions on T1-weighted images. While more tailored to CNS tumors than RECIST, the Macdonald criteria suffered from a heavy reliance on contrast enhancement and lacked consideration of non-enhancing lesions (NELs) or clinical deterioration.

The RANO group proposed updated criteria in 2010 [4]. The RANO criteria incorporated several additional elements, including two-dimensional measurement of contrast-enhancing lesions, qualitative assessment of NELs on T2/FLAIR images, clinical status, and steroid dose. Since then, the RANO framework has expanded into a range of subtype-specific criteria (Table 1) through various efforts [13,14], including adaptations for immunotherapy [5], brain metastases (RANO-BM) [15], and low-grade gliomas (RANO-LGG) [16]. Recently, in 2023, RANO 2.0 [17] was introduced as a further refinement of this evolving framework, incorporating features such as the definition of minor response, refined guidance for new-lesion assessment, and enhanced applicability in the era of multimodal therapy and immune checkpoint inhibitors. Nevertheless, the adoption of these tools remains inconsistent across trials, and uniform implementation in trial design is still lacking.

### 1.3. Why Consensus Is Needed Now More than Ever

Recent advances in oncology drug development have increased the need for flexible but reproducible efficacy assessment frameworks. In rare cancers and molecularly defined subtypes, development strategies increasingly rely on international collaboration and early-phase efficacy signals, making consistent endpoint definitions essential for scientific validity and regulatory interpretability [1,19,20]. However, the neuro-oncology field continues to face challenges due to the lack of universally accepted standards for efficacy endpoints [21]. Eisele et al. stated in their review that one of the key aspects in this regard is the development of objective and standardized criteria that allow for accurate response assessment in clinical trials and prevent the misclassification of responders and non-responders [22]. This uncertainty has become a barrier to clinical development, affecting not only the design and interpretation of Phase III trials but also the appropriateness of accelerated approval pathways. Consequently, there is a growing need for scientific consolidation and international standardization of assessment criteria in CNS tumor trials. A renewed focus on this issue is essential to ensure that novel therapies can be developed, evaluated [23], and approved based on robust and harmonized efficacy metrics.

### 1.4. Objectives of This Review

This narrative review aims to elucidate the current landscape and challenges associated with the selection of efficacy endpoints in Phase II clinical trials for CNS tumors, with a particular focus on GBM and malignant and/or recurrent meningioma. Although brain metastases and pediatric CNS tumors are not the primary focus of the empirical analyses incorporated in this review, relevant response assessment frameworks are discussed when they inform the broader landscape of endpoint harmonization in neuro-oncology. By outlining the historical evolution of brain tumor–specific imaging response criteria and the introduction of RANO 2.0 as an evolving framework, we critically examine the validity, reproducibility, and international harmonization of efficacy endpoints. Unlike prior reviews that have primarily focused on the development and application of individual response criteria, this review integrates regulatory science perspectives, previously published empirical analyses of Phase I and Phase II CNS tumor trials, and emerging endpoint frameworks to clarify how endpoint strategies can be harmonized across trial design, imaging assessment, and regulatory interpretation. In particular, we emphasize the interface between clinical decision-making and regulatory endpoint evaluation, highlighting the need for alignment between trial-based efficacy measures and real-world treatment considerations.

## 2. Methods

The review purposefully draws upon key references published over the past two decades, including consensus guidelines such as the RANO and RANO 2.0 criteria, the 2021 World Health Organization (WHO) Classification of CNS Tumors [24], major clinical trials, and regulatory documents issued by agencies such as the Food and Drug Administration (FDA), European Medicines Agency (EMA), and Pharmaceuticals and Medical Devices Agency (PMDA). This study was conducted as a narrative review intended to provide a regulatory science-oriented synthesis rather than a formal systematic review. The literature search primarily utilized PubMed and ClinicalTrials.gov, supplemented by official regulatory documents and consensus statements. Search terms included combinations of keywords such as “brain tumor,” “glioblastoma,” “meningioma,” “Phase I,” “Phase II,” “efficacy endpoint,” “RANO,” “RANO 2.0,” “response assessment,” and “clinical trial”. Priority was given to influential consensus papers, pivotal clinical trials, and representative studies that contributed substantially to the development, validation, or regulatory interpretation of efficacy endpoints in neuro-oncology. Studies were included if they addressed response assessment criteria, efficacy endpoint selection, trial design, or regulatory evaluation relevant to CNS tumor clinical trials. Studies unrelated to CNS tumors, non-clinical experimental studies, and articles without relevance to efficacy endpoint assessment were not prioritized. Because this was not a formal systematic review, the selection of studies was not intended to be exhaustive, and the review focuses on representative studies most relevant to endpoint harmonization and regulatory interpretation. In addition to the narrative literature synthesis, this review incorporates previously published empirical analyses conducted by our group regarding Phase I and Phase II CNS tumor trials in neuro-oncology.

## 3. Current Challenges in Efficacy Assessment for CNS Tumors

### 3.1. Principles and Limitations of RECIST in Neuro-Oncology

**Figure 1:**
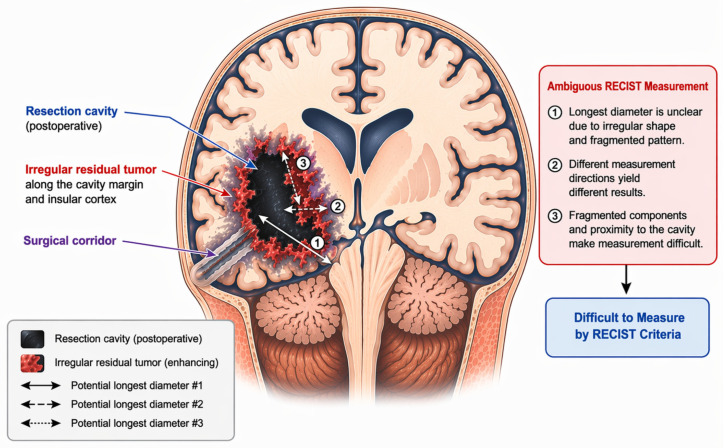
Challenges in applying RECIST criteria to postoperative residual brain tumors. Following surgical resection, residual tumors often exhibit irregular and fragmented morphology along the resection cavity. Tumor extension may be influenced by cortical sulcal structures and white-matter tract anatomy, resulting in complex non-spherical geometries that are difficult to characterize using unidimensional measurements. In addition, postoperative cavity deformation and the close proximity of residual tumor to the resection margin further complicate boundary delineation. These features highlight the limitations of the RECIST criteria in accurately assessing treatment response in neuro-oncology. RECIST: Response Evaluation Criteria in Solid Tumors.

RECIST 1.1 defines tumor response primarily through unidimensional measurement of selected target lesions and changes in the sum of their longest diameters [2,3,25]. Although this framework provides a simple and reproducible approach for many extracranial solid tumors, it assumes that tumor burden can be captured using well-demarcated, measurable lesions [26,27]. In contrast to many extracranial solid tumors, gliomas often exhibit highly irregular and infiltrative growth patterns. Tumor spread tended to be constrained by cortical sulcal structures and frequently followed white-matter tracts, resulting in complex, non-spherical geometries that challenge unidimensional measurement. Key limitations include the need to interpret both contrast-enhanced T1-weighted and T2/FLAIR images, the frequent presence of NELs, irregular or fragmented postoperative residual disease [28,29] (Figure 1), treatment-related imaging changes such as pseudoprogression [10,30] and radiation necrosis [9,31], and the unique functional constraints imposed by the fixed intracranial compartment. A prominent example is seen in trials of bevacizumab for GBM. Although imaging assessments often showed marked reductions in contrast enhancement and PFS-related improvement, these effects did not translate into a consistent OS benefit [32]. In such cases, disappearance of enhancement does not necessarily equate to true tumor shrinkage, underscoring the unique imaging behavior of CNS tumors.

Nevertheless, RECIST may retain limited utility in selected CNS-related trial contexts, such as brain metastases or clearly measurable enhancing lesions, particularly when CNS lesions are evaluated within broader systemic solid tumor trials. Thus, the limitation of RECIST in neuro-oncology should be understood as context-dependent rather than absolute.

### 3.2. Imaging Confounders in Neuro-Oncology Endpoint Assessment

Pseudoprogression refers to transient post-treatment increases in lesion size or enhancement, particularly after radiotherapy with temozolomide, driven by vascular permeability, inflammatory changes, and blood–brain barrier disruption rather than true tumor growth [8,33]. In GBM treatment, pseudoprogression typically occurs within the first three months post-treatment and has been reported in approximately 10–30% of cases [34]. Misclassifying pseudoprogression as true progression may lead to premature discontinuation of an otherwise effective investigational therapy, thus compromising the trial’s evaluative power and potentially leading to false-negative conclusions. Under the iRANO criteria, radiographic progression occurring within six months of immunotherapy initiation in clinically stable patients should be confirmed on follow-up imaging after approximately three months [5], although this delayed-confirmation approach may complicate trial efficiency, endpoint interpretation, and single-arm trial design. Radiation necrosis is a delayed treatment-related injury characterized by vascular damage, hypoxia, and necrotic degeneration, often occurring months after radiotherapy [9,31]. NELs represent another major challenge. In LGG and recurrent GBM, tumor burden may be reflected primarily by T2/FLAIR hyperintensity rather than T1 contrast enhancement, representing a mixture of edema, infiltrative tumor, and reactive changes [35]. Although frameworks such as RANO-LGG [16] and Advanced RANO incorporate T2/FLAIR-based assessment, standardized implementation, reproducibility, and correlation with clinical outcomes remain unresolved.

### 3.3. Limitations of Conventional ORR in Neuro-Oncology

Findings from our previous regulatory science analyses highlighted several critical limitations [21,29,36]. First, many CNS-directed therapies do not produce measurable tumor shrinkage on imaging, despite having meaningful clinical activity. In GBM immunotherapy, complex imaging patterns and delayed radiographic responses have been widely reported, making conventional ORR-based assessment insufficient to capture true therapeutic benefit [5,8,9,34]. Second, in our comprehensive review of Phase II GBM trials conducted between 2020 and 2022 [29], we found that only 7% of studies used ORR as a primary endpoint. The majority favored PFS or OS, reflecting a growing recognition of ORR’s limitations. Moreover, among the trials that did employ ORR, many lacked clear definitions—even when utilizing RANO or iRANO criteria—indicating a lack of consistency in how response assessments are operationalized across studies. Third, in our regulatory science study of Phase II meningioma trials [21], we observed that very few patients achieved partial response (PR)/complete response, underscoring the limited sensitivity of ORR in this tumor type. In high-grade or treatment-refractory meningiomas (WHO grades 2–3), disease stabilization over a prolonged period is often regarded as a meaningful therapeutic outcome. In such cases, alternative endpoints such as Disease Control Rate (DCR) or 6-month PFS (PFS-6) may better reflect clinical benefit.

## 4. Evolution of Endpoint Strategies in Neuro-Oncology Clinical Trials

### 4.1. From Original RANO to RANO 2.0: A Refined Framework

The RANO criteria were introduced in 2010 as a brain tumor–specific framework that incorporated enhancing disease, non-enhancing lesions, corticosteroid use, and clinical status into response assessment. However, increasing use of immunotherapy, anti-angiogenic agents, molecularly defined tumor classification, and heterogeneous trial designs exposed limitations in the consistency and operationalization of RANO-based assessments. Thus, the RANO 2.0 criteria [17] were introduced as an updated framework intended to refine and harmonize response assessment across contemporary adult glioma trials. Rather than representing a definitive solution, it should be understood as an evolving framework whose implementation requires further validation, protocol-level standardization, and careful regulatory interpretation (Table 2).

### 4.2. RANO 2.0: Conceptual Advances and Practical Implications

#### 4.2.1. Unification of Response Criteria Across Glioma Subtypes

A central feature of RANO 2.0 is its attempt to promote greater consistency in response assessment across glioma subtypes. Rather than maintaining separate criteria for high-grade and LGGs, RANO 2.0 proposes a single evaluation framework applicable across molecularly defined glioma entities. This approach is intended to accommodate tumors characterized by predominantly enhancing lesions, NELs, or mixed imaging patterns. From a regulatory science perspective, this unification may improve cross-trial comparability and may align response assessment with the molecular classification paradigm that now underpins clinical development in neuro-oncology, provided that implementation is standardized across protocols and institutions.

#### 4.2.2. Redefinition of Baseline Imaging

RANO 2.0 recommends redefining the baseline imaging time point for newly diagnosed gliomas. Specifically, the post-radiotherapy MRI, rather than the immediate postoperative scan, is designated as the reference baseline. This change reflects evidence that early postoperative imaging is frequently confounded by surgical artifacts, hemorrhage, and inflammatory changes, whereas post-radiotherapy imaging provides a more stable reference for longitudinal assessment. Moreover, analyses have demonstrated improved correlation between PFS and OS when progression is defined relative to the post-radiotherapy baseline, supporting the potential clinical relevance of this approach while underscoring the need for prospective validation in trial settings.

#### 4.2.3. Measurement Methodology: Two-Dimensional Assessment and the Role of Volumetry

RANO 2.0 retains 2D measurements as the standard methodology for tumor assessment, thereby preserving continuity with prior RANO criteria and ensuring feasibility in multicenter trials. At the same time, volumetric assessment is permitted as an optional approach when predefined quality and reproducibility requirements are met. Thresholds for response and progression were recalibrated to maintain consistency between 2D and volumetric measurements. This pragmatic compromise acknowledges the theoretical advantages of volumetric analysis while recognizing that standardized implementation remains challenging in routine clinical trials.

#### 4.2.4. Approach to Pseudoprogression and Confirmation of Progression

**Figure 2:**
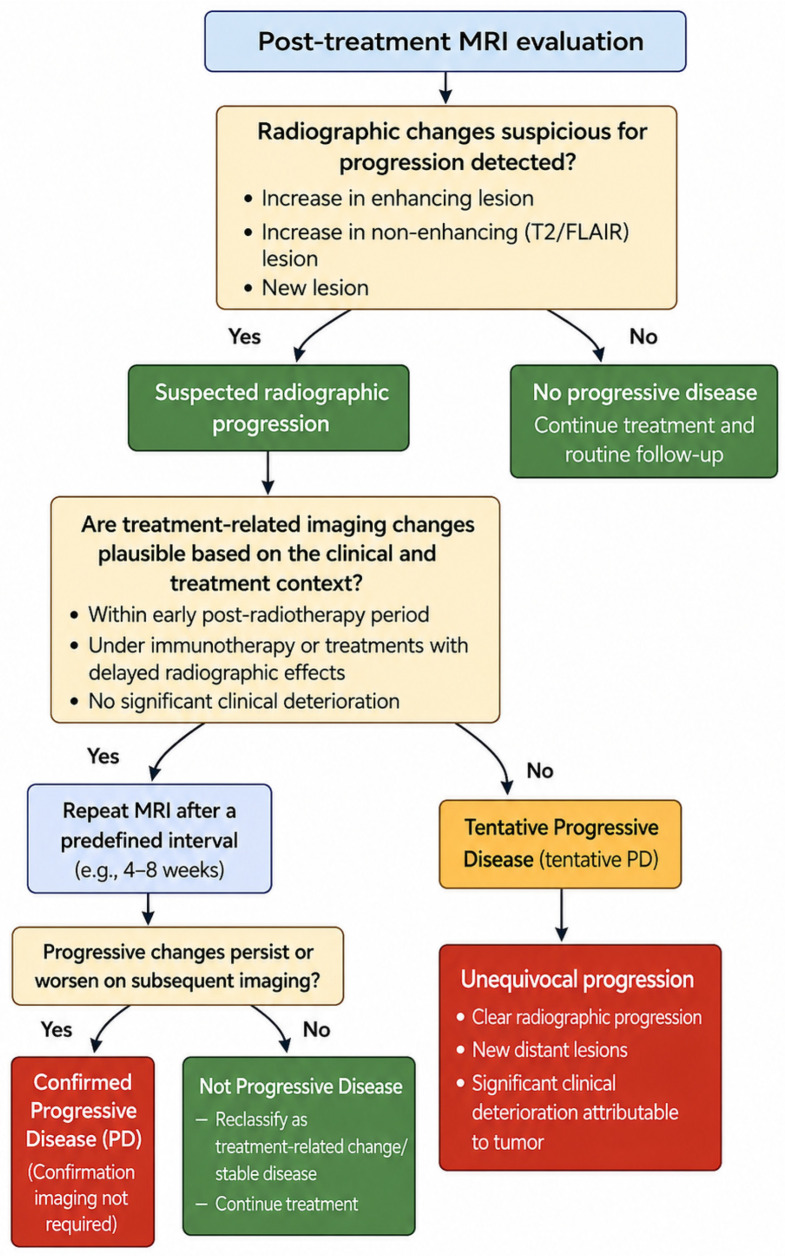
Flowchart for progression assessment according to RANO 2.0. The figure summarizes the recommended approach to suspected radiographic progression, particularly within the early post-radiotherapy period. Progression suspected within 12 weeks after completion of radiotherapy should be interpreted cautiously, and follow-up imaging may be required to distinguish true progression from treatment-related changes when the patient is clinically stable. MRI; magnetic resonance imaging, PD; progressive disease.

The handling of pseudoprogression represents an important area of refinement in RANO 2.0 (Figure 2). Assessment of progression within the first 12 weeks following completion of radiotherapy is particularly challenging because of the high incidence of pseudoprogression, and imaging findings during this period should therefore be interpreted with caution. Follow-up imaging is often necessary to distinguish true progression from treatment-related effects. This approach contrasts with earlier frameworks that mandated confirmation in a broader range of settings and reflects an effort to balance diagnostic rigor against the risk of delaying appropriate clinical decision-making.

#### 4.2.5. Reconsideration of Non-Enhancing Progression in GBM

In IDH-wildtype GBM, RANO 2.0 substantially revises the role of non-enhancing progression assessed on T2/FLAIR imaging. Based on evidence demonstrating limited correlation with OS, non-enhancing progression alone is generally no longer considered sufficient for defining progression in this population. This represents an important modification of earlier criteria and reflects a data-driven reassessment of the clinical relevance of T2/FLAIR changes in aggressive gliomas.

#### 4.2.6. Integration of Clinical Status and Neurological Assessment

RANO 2.0 emphasizes that imaging findings should not be interpreted in isolation. Deterioration in clinical or neurological status, when attributable to tumor progression rather than alternative causes, may support a progression designation even in the absence of unequivocal radiographic change. While structured neurological scales such as the Neurologic Assessment in Neuro-Oncology (NANO) scale [37] are discussed as promising adjuncts, their routine integration into response criteria is deferred pending further validation.

#### 4.2.7. Regulatory Science Perspective

From a regulatory science standpoint, RANO 2.0 represents an implementation-oriented evolution rather than a definitive endpoint. The framework seeks to harmonize scientific validity, feasibility in multicenter trials, and consistency in regulatory interpretation. Importantly, RANO 2.0 is positioned as a living framework, designed to accommodate future refinements as imaging technologies, therapeutic modalities, and biomarker strategies continue to evolve. Overall, RANO 2.0 may serve as a useful and pragmatic response assessment framework that reflects contemporary neuro-oncology practice and attempts to address longstanding limitations in efficacy evaluation, while acknowledging the need for ongoing refinement to support robust regulatory decision-making.

#### 4.2.8. Remaining Challenges and Unresolved Issues in RANO 2.0

RANO 2.0 should be viewed as a promising but still evolving framework rather than a definitive solution. Several practical issues remain unresolved, including the assessment of multifocal or leptomeningeal disease, the reproducibility of minor response and non-enhancing lesion evaluation, and the standardized implementation of volumetric assessment. Although formal interobserver agreement data specifically for RANO 2.0 remain limited, prior studies of RANO-based assessments have highlighted measurement variability, particularly for non-enhancing or infiltrative lesions. In multicenter trials, variability in imaging acquisition, lesion segmentation, local versus central review, missing imaging data, and early discontinuation may affect endpoint reliability. In addition, regulatory acceptance of RANO 2.0-based endpoints will likely depend on disease context, mechanism of action, endpoint definition, and the robustness of prespecified imaging review procedures. These considerations highlight the need for prospective protocol-level standardization and further validation of RANO 2.0 in clinical trial settings.

### 4.3. Harmonization with the 2021 WHO CNS Tumor Classification

The 5th edition of the WHO Classification of Tumors of the Central Nervous System [24] marked a paradigm shift in neuro-oncology by incorporating molecular diagnostics—such as IDH mutation, 1p/19q codeletion, TERT promoter mutations, and MGMT promoter methylation—into the histopathological framework. This redefined tumor entities and grading schemes across adult and pediatric CNS tumors. For example, IDH-wildtype gliomas with molecular features such as EGFR amplification or chromosome 7 gain/10 loss are now classified as GBM, regardless of their histologic appearance. In pediatric oncology, entirely new molecular entities have been introduced, reinforcing the necessity for molecularly stratified trial design and efficacy evaluation. This evolution underscores the fact that tumors sharing the same histological type can vary substantially in prognosis and treatment response based on their genetic profiles. Consequently, molecular classification has become essential in both patient stratification and endpoint interpretation.

For example, IDH-mutant low-grade gliomas often demonstrate slow, infiltrative, and predominantly non-enhancing growth patterns, making conventional contrast-enhancement-based response criteria less informative. In such settings, progression-free survival and longitudinal T2/FLAIR-based assessments may provide more clinically meaningful measures of therapeutic benefit than objective response rate alone. Conversely, MGMT promoter-methylated glioblastomas are associated with higher rates of treatment-related imaging changes, including pseudoprogression, which may complicate early radiographic response interpretation following chemoradiotherapy. These examples illustrate that molecularly defined CNS tumor subtypes may require distinct endpoint strategies and response assessment frameworks. The recent vorasidenib trial in IDH-mutant low-grade glioma illustrates this paradigm shift, showing that molecularly defined tumors may require endpoint strategies that differ from those used in rapidly progressive enhancing gliomas. In this setting, treatment benefit may be captured more appropriately by progression-free survival, longitudinal T2/FLAIR-based assessment, and growth-kinetic changes than by conventional contrast-enhancement-based ORR alone.

From the standpoint of harmonization with RANO 2.0, the WHO CNS 2021 framework implies that efficacy assessment may extend beyond morphologic changes (e.g., size and enhancement patterns) to include molecular biomarkers and functional imaging (such as PET tracers or liquid biopsy markers). Future clinical development strategies will need to adopt integrative approaches that align radiological, molecular, and clinical endpoints in a cohesive framework.

### 4.4. Recent Trends in Endpoint Strategies in Early-Stage Clinical Trials for CNS Tumors

#### 4.4.1. Phase II GBM Trials

**Figure 3:**
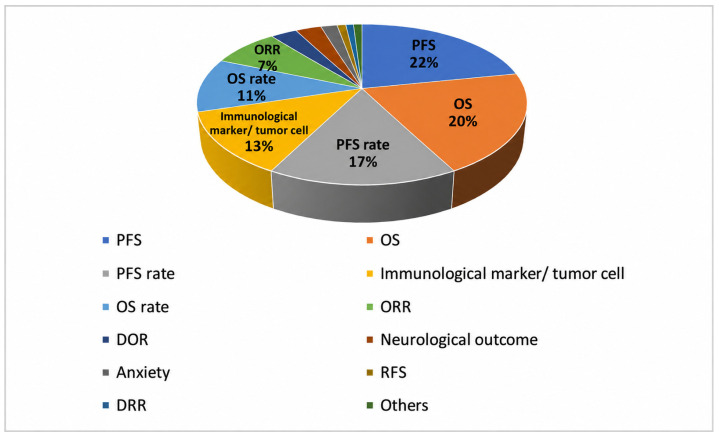
Trends in efficacy endpoint selection in Phase II glioblastoma trials. Data are summarized from our previously published analysis [29]. PFS; progression-free survival; OS; overall survival; ORR; objective response rate; DOR; duration of response; RFS; recurrence-free survival; DRR; durable response rate.

GBM remains one of the most treatment-resistant and lethal tumors of the CNS, posing significant challenges in drug development. In particular, the selection of efficacy endpoints in Phase II trials is a pivotal factor that can influence the success of subsequent Phase III studies and regulatory approval. Against this backdrop, our previously published cross-national analysis evaluated recent Phase II clinical trials targeting GBM [29]. Among 88 trials, PFS, OS, and PFS rates were the most common primary endpoints at 22 (22%), 20 (20%), and 17 (17%), respectively. Many studies employed multiple co-primary or composite endpoints, and several immunotherapy trials incorporated the iRANO criteria. These findings suggest that PFS and related time-to-event endpoints remain dominant, while ORR continues to be used in a subset of trials (Figure 3). This analysis provides empirical support for understanding the current status and future directions of endpoint standardization in the RANO 2.0 era.

#### 4.4.2. Phase II Meningioma Trials

A comprehensive review of efficacy endpoints used in pharmacological trials for surgery- and radiotherapy-resistant meningiomas was published in 2014 [38]. When analyses were conducted to define outcome benchmarks, the only endpoint that could be consistently extracted across studies was the 6-month PFS (PFS-6) rate. Based on these findings, the review concluded that PFS-6 should be recommended as a benchmark endpoint for subsequent clinical trials. However, even by 2019, no standardized criteria existed for assessing response or progression in clinical trials involving meningioma patients. In response to these challenges, the RANO Meningioma Working Group proposed specific recommendations regarding response criteria and efficacy endpoints for clinical trials in patients with meningioma [39].

To clarify the current status of efficacy endpoint selection, our previously published cross-national analysis evaluated Phase II Meningioma Trials in PubMed from April 2001 to March 2021, identifying 48 eligible trials [21]. Our review found that PFS rate was the most frequently used primary endpoint, appearing in 38% of trials; PFS-6, PFS-12, and PFS-36 were used as primary endpoints in 31%, 4%, and 2% of trials, respectively. This was followed by ORR (33%), PFS (22%), and OS (2%). Importantly, there was substantial variation in how these endpoints were defined, with a mix of assessment criteria including RANO, RECIST, and WHO guidelines. PFS and related time-to-event endpoints remain dominant, while ORR continues to be used in a subset of trials. Given the rarity of high-grade or treatment-refractory meningioma and the difficulty in enrolling large cohorts, the establishment of rational, harmonized endpoint strategies is crucial—especially in the context of international collaborative trials or conditional approval pathways. Moving forward, it will be essential to reconstruct evaluation frameworks that align with the biological characteristics and natural history of meningioma.

#### 4.4.3. Exploratory Efficacy Assessments in Phase I Trials for CNS Tumors

With the advancement of molecular targeted therapies and immunotherapies, there has been a growing trend toward incorporating exploratory efficacy assessments into Phase I trials for CNS tumors, in addition to traditional dose-finding objectives. To elucidate current practices, our previously published cross-national analysis evaluated 42 Phase I trials focused specifically on brain tumors [36]. For analytical purposes, trials were stratified into two categories: those exclusively enrolling patients with brain tumors (Group A) and those including brain tumors within broader solid tumor cohorts (Group B). Across the included studies, the median number of primary endpoints was 1.5 (range: 1–6), while the median number of secondary endpoints was 5 (range: 0–19). Efficacy-related endpoints were specified as primary endpoints in 2 trials (5%) and as secondary endpoints in 31 trials (78%). Among these 31 trials, a total of 94 efficacy endpoints were identified, comprising ORR (*n* = 24), PFS (*n* = 22), OS (*n* = 20), duration of response (DOR; *n* = 9), and DCR (*n* = 8). Notably, although ORR was assessed using multiple evaluation frameworks, RECIST-based assessment was significantly less frequently applied in trials limited to brain tumors (Group A) compared with those including mixed solid tumor populations (Group B). Overall, recent Phase I trials have increasingly incorporated efficacy endpoints as secondary objectives. Approximately half of the trials included ORR, PFS, or OS, while around one-quarter incorporated DOR or DCR (Table 3). This lack of consistency may impact downstream clinical trial design. The use of CNS-appropriate evaluation criteria from the exploratory phase onward could help create a more seamless and scientifically sound bridge to Phase II and III trials.

## 5. Regulatory Science Approach to Endpoint Harmonization

### 5.1. Significance of Endpoint Strategy Standardization: Scientific Validity, Reproducibility, and Global Comparability

**Figure 4:**
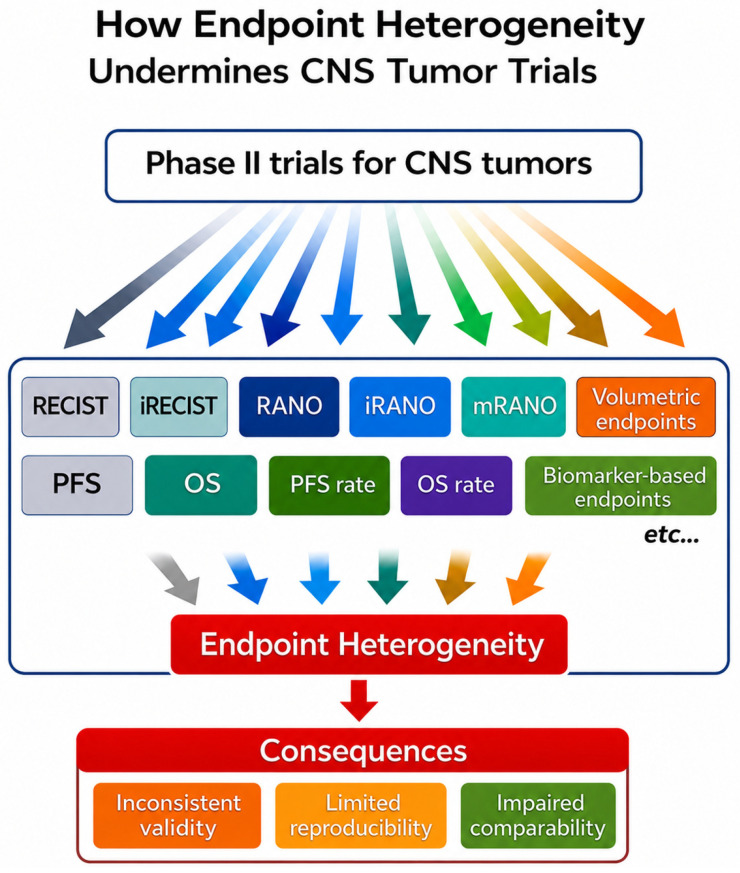
**Overview of endpoint heterogeneity in CNS tumor trials.** The figure summarizes how heterogeneity in response assessment frameworks and efficacy endpoints may affect trial design, endpoint interpretation, reproducibility, cross-study comparability, and regulatory evaluation. The endpoint landscape includes RANO-based frameworks, modified RECIST, iRECIST, PET RANO 1.0, volumetric assessment, growth-kinetic approaches, and PFS2, depending on tumor biology and therapeutic context. RECIST; Response Evaluation Criteria in Solid Tumors; RANO; Response Assessment in Neuro-Oncology; iRANO; immunotherapy Response Assessment in Neuro-Oncology; iRECIST; immunotherapy Response Evaluation Criteria in Solid Tumors; PET; positron emission tomography; PFS; progression-free survival; PFS2; second progression-free survival; OS; overall survival.

Standardizing efficacy endpoints in neuro-oncology is essential for ensuring comparability across international trials [40]. However, applying endpoint frameworks designed for other solid tumors without adequate adaptation may compromise the scientific validity of efficacy assessments (Figure 4). CNS tumors—despite being classified as solid tumors—often require unique evaluation methodologies due to their atypical imaging features and post-treatment dynamics. This has led to increasing recognition that brain tumors require tumor-specific evaluation frameworks rather than direct application of conventional solid tumor criteria. In this context, the development and dissemination of tumor-specific response assessment frameworks are critical from a regulatory science perspective.

In high-grade CNS malignancies such as GBM, the choice of efficacy endpoint can significantly influence the interpretation of therapeutic outcomes. Nevertheless, current Phase II studies frequently employ a variety of criteria, including RECIST, RANO, iRANO, mRANO, and volumetric response assessment [41,42]. There is also substantial heterogeneity in the definitions and measurement schedules for common endpoints such as ORR and PFS. This heterogeneity not only reduces reproducibility and comparability across trials but also creates challenges in designing global clinical trials and in regulatory decision-making.

Importantly, the need for endpoint harmonization is not supported solely by our empirical analyses. Independent international groups have also highlighted substantial heterogeneity in neuro-oncology trial design and response assessment. The RANO group emphasized the need to reconsider Phase II trial designs and endpoint selection in neuro-oncology [14], while RANO-based reviews and recommendations for meningioma and brain metastases have similarly noted variability in response criteria, progression definitions, and endpoint selection [15,38,39]. In pediatric CNS tumors, the RAPNO working group has also developed tumor-specific response assessment recommendations, underscoring that endpoint standardization requires disease- and population-specific frameworks [43,44]. Harmonized endpoints facilitate cross-trial meta-analyses, integration with real-world data, and overall transparency and robustness in benefit–risk evaluations. Frameworks such as RANO 2.0 may contribute to this goal, but their regulatory utility depends on continued validation and standardized implementation.

### 5.2. Advantages and Considerations of Adopting RANO 2.0

While the adoption of RANO 2.0 may provide several important advantages in clinical trials, it also requires careful implementation. RANO 2.0 aims to provide a more unified framework for response assessment across enhancing tumors and, in selected contexts, non-enhancing disease. The introduction of minor response may help capture intermediate treatment effects that would previously have been classified as stable disease, although its usefulness depends on tumor characteristics and imaging context [17]. RANO 2.0 also introduces more cautious handling of early radiographic worsening and incorporates neurological stability alongside imaging findings, supporting a more clinically grounded assessment. The formal consideration of NELs is conceptually important, but reproducible implementation remains challenging.

Despite these advantages, RANO 2.0 introduces operational complexity. Accurate assessment requires integration of imaging findings, NEL evaluation, corticosteroid use, neurological status, and, when applicable, volumetric measurements. Therefore, trained personnel, harmonized imaging protocols, and robust review procedures are necessary to ensure consistency and reproducibility. In single-arm or multicenter trials, independent central review may be particularly important to reduce assessment bias and improve regulatory interpretability.

### 5.3. Clinical Trial Design and Regulatory Evaluation

Regulatory agencies such as PMDA, FDA, and EMA have long assessed clinical trial designs through scientific consultations and guidance documents, emphasizing endpoint validity, interpretability, and clinical relevance. Our comprehensive review of Phase II GBM trials revealed that, while endpoints such as RECIST and RANO remain widely used, their implementation and interpretation vary significantly across studies, raising concerns about consistency and comparability from a regulatory standpoint [29]. In particular, ORR—when assessed solely based on contrast enhancement—may be prone to overestimation due to phenomena such as “pseudo-response” observed with immunotherapy or anti-angiogenic agents. Therefore, the validity and interpretability of efficacy endpoints remain central considerations in oncology drug development. Novel criteria such as iRECIST [45] and other immunotherapy-optimized assessment methods have also been introduced, reflecting a broader trend toward balancing scientific rigor with regulatory flexibility—even in CNS tumor trials. These developments may suggest that endpoint selection in trial design should go beyond simple adherence to existing guidelines. The choice of response assessment framework may affect expected response or progression rates and, consequently, sample size assumptions in Phase II trials. Therefore, sponsors should strategically select endpoints that align with tumor biology, mechanism of action, and regulatory purpose, while prospectively defining imaging schedules, missing-data handling, early discontinuation rules, and the use of local versus independent central review.

### 5.4. Emerging Endpoint Frameworks and Operational Considerations

Beyond MRI-based RANO frameworks, several emerging endpoint strategies may further refine efficacy assessment in CNS tumor trials. PET RANO 1.0 has recently been proposed to standardize amino acid PET-based response assessment for diffuse gliomas, potentially capturing metabolic tumor activity that may not be fully characterized by conventional MRI; however, further validation is needed to define its relationship with MRI-based RANO 2.0, clinical outcomes, and regulatory decision-making [46]. Volumetric assessment and growth-kinetic approaches may also be useful, particularly for infiltrative or slowly progressive tumors such as IDH-mutant low-grade gliomas, but they require standardized segmentation, imaging acquisition, software platforms, and reproducibility assessment. Similarly, PFS2 may provide information on the durability of disease control beyond first progression, although its role as a surrogate endpoint in neuro-oncology remains insufficiently established [47]. From a trial-design perspective, the choice of response framework may affect sample size assumptions, endpoint event rates, missing-data handling, and the need for local versus central review. Therefore, endpoint harmonization requires not only selecting an appropriate response criterion but also prospectively standardizing how the endpoint is measured, analyzed, and interpreted.

## 6. Future Directions and Recommendations

### 6.1. Recommendations for Endpoint Design in Phase I and II Trials

In early-phase clinical trials for brain tumors, careful selection of efficacy endpoints is necessary. Specific challenges—such as the interpretation of NELs, differentiation of true progression from treatment-related changes—require tailored approaches. As such, reliance solely on tumor shrinkage or ORR may be insufficient, and the incorporation of time-to-event endpoints (e.g., PFS, time to progression, DOR) is more appropriate in practical terms. In Phase II trials, even single-arm studies should ideally adopt prospectively defined, CNS tumor–appropriate endpoints, which may include RANO 2.0-compliant endpoints when appropriate, as benchmarks. While many prior studies used PFS-6 based on the original RANO criteria as a primary endpoint, the introduction of RANO 2.0 expands the available framework alongside immunotherapy-adapted frameworks such as iRANO. These multidimensional assessment strategies may help improve future comparability across trials. In parallel, when generating evidence from real-world data in unblinded settings, trials should incorporate clearly defined RANO 2.0-based evaluation criteria. Reporting should adhere to structured formats—including standardized tables and explicit definitions.

Importantly, as RANO 2.0 and other evolving frameworks are not yet universally recognized as “gold standards,” the use of multiple co-primary or secondary endpoints (e.g., ORR, PFS, clinical benefit rate) should be acceptable. Such multi-endpoint designs, supplemented with sensitivity analyses and additional exploratory indicators, enhance the robustness and interpretability of trial findings. In recent years, a “Phase II skip” development strategy has illustrated the possibility of streamlined development in selected molecularly defined glioma settings, exemplified by the INDIGO trial [48,49]—a Phase III study of vorasidenib in patients with IDH-mutant low-grade glioma that proceeded without a preceding Phase II trial. In such strategies, rigorous scientific rationale, well-defined disease biology, the validity of surrogate endpoints, and—above all—the reliability of standardized assessment criteria are critically important for regulatory decision-making. Particularly in gliomas, where conventional evaluation metrics are often insufficient, the implementation of novel assessment frameworks such as RANO 2.0 may contribute to accelerated development pathways that bypass Phase II trials, provided that their use is prospectively defined and appropriately validated. Moreover, because Phase III confirmatory trials are frequently impractical in neuro-oncology, decision-making often depends on Phase II—or even Phase I—data. This underscores the urgent need to advance endpoint standardization in early-phase brain tumor trials.

### 6.2. Challenges and Outlook for the Dissemination of RANO 2.0

RANO 2.0 represents one of the most recent efforts to update the response assessment framework in CNS tumor evaluation. However, several practical and educational hurdles remain for its widespread adoption. Standardizing imaging interpretation and ensuring reproducibility remain the core challenges—particularly in non-specialist institutions or real-world clinical environments where familiarity with RANO 2.0 may be limited. The implementation of centralized review systems, including Independent Review Committees, is further constrained by resource and personnel demands. Additionally, RANO 2.0-compliant data collection and analysis require harmonization of imaging protocols and report formats. Despite these challenges, broader adoption of RANO 2.0 may contribute to international harmonization in clinical trials and regulatory evaluation, provided that its implementation is supported by standardized imaging protocols, training, and independent review procedures. Multicenter and multinational data collection using harmonized endpoint definitions will be important for validating the practical utility of RANO 2.0 and other evolving response assessment frameworks.

## 7. Conclusions

This narrative review does not aim to propose a single definitive efficacy endpoint, but rather to provide a regulatory science framework for understanding endpoint selection in CNS tumor trials. The central message is that CNS tumor trials require disease-specific endpoint strategies that account for anatomical, radiographic, biological, and treatment-related complexities. Conventional solid tumor metrics, particularly RECIST-based response assessment, have limited applicability in many CNS tumor settings, whereas RANO 2.0 represents an important but still evolving framework for harmonized response assessment.

Future trials should prospectively define imaging protocols, response and progression criteria, confirmation rules, missing-data handling strategies, and independent review procedures when appropriate. In parallel, radiologic, clinical, molecular, and patient-centered endpoints should be integrated to improve scientific validity, reproducibility, cross-trial comparability, and regulatory interpretability. Endpoint harmonization should therefore be understood not simply as selecting a response criterion, but as standardizing the entire endpoint strategy from trial design to regulatory evaluation.

## Figures and Tables

**Table 1:** Evolution and Key Features of Brain Tumor-Specific Imaging Response Criteria.

Name	Primary Target	Key Features
Macdonald criteria (1990) [12]	High-grade gliomas (HGG)	Bidimensional (2D) assessment of contrast-enhancing lesions; limited consideration of NELs
RANO (2010) [4]	HGG	Integrated assessment of contrast-enhancing/NELs (T2/FLAIR), clinical status, and corticosteroid use
RANO-LGG (2011) [16]	Low-grade gliomas (LGG)	Focus on NELs; T2/FLAIR-based assessment framework
iRANO (2015) [5]	Patients with glioma receiving immunotherapy	For radiographic PD within 6 months after immunotherapy initiation, confirmation on follow-up imaging is recommended in clinically stable patients
RANO-BM (2015) [15]	Brain metastases	Designed to evaluate treatment-related changes after local therapies (stereotactic radiosurgery or whole-brain radiotherapy)
mRANO (2017) [18]	HGG, including bevacizumab-treated cases	Emphasizes “minor response” and treatment durability; redefinition of PD in anti-VEGF settings
RANO 2.0 (2023) [17]	Adult diffuse glioma trials across molecular subtypes	Explicit criteria for minor response; updated rules for new contrast-enhancing lesions; clearer category definitions

RANO; Response Assessment in Neuro-Oncology; HGG; high-grade glioma; NEL; non-enhancing lesion; FLAIR; fluid-attenuated inversion recovery; LGG; low-grade glioma; iRANO; immunotherapy Response Assessment in Neuro-Oncology; PD; progressive disease; BM; brain metastasis; VEGF; vascular endothelial growth factor.

**Table 2:** Comparison of response criteria.

Criteria	Enhancing Lesion	NEL	Steroid	Clinical Status	Confirmation Required	Target Population
Macdonald criteria [12]	2D measurement of contrast-enhancing lesions	Not considered	Required for response interpretation	Limited consideration	Not required	Adults with HGG treated with cytotoxic therapies, primarily in the pre-temozolomide era
RECIST 1.1 [2]	Unidimensional longest diameter measurement of enhancing lesions	Not considered	Not considered	Not considered	Required in some settings	Non-CNS solid tumors with measurable, well-demarcated lesions; not originally designed for primary brain tumors
RANO [4]	2D measurement of enhancing lesions with contextual interpretation	Qualitative assessment of T2/FLAIR changes	Integrated into response determination	Explicit integration of neurological status	Context-dependent	Adult patients with HGG undergoing surgery, radiotherapy, and systemic therapy
RANO-LGG [16]	Not primary focus (enhancement often absent)	Primary endpoint based on T2/FLAIR abnormalities	Considered contextually	Integrated	Often required	Patients with diffuse LGG in whom non-enhancing tumor components predominate
iRANO [5]	Same as RANO, with delayed confirmation for early progression	Qualitative assessment with delayed progression confirmation	Integrated with immune-related context	Integrated, particularly for delayed response	Confirmation is recommended for early radiographic progression in clinically stable patients	Patients with CNS tumors treated with immunotherapies, particularly immune checkpoint inhibitors
RANO-BM [15]	2D measurement adapted for post-radiotherapy metastases	Limited consideration	Considered variably	Integrated	Context-dependent	Patients with brain metastases receiving radiotherapy and/or systemic anticancer treatments
mRANO [18]	2D measurement with emphasis on minor response and durability	Qualitative assessment, context-dependent	Integrated with emphasis on anti-VEGF effects	Integrated	Often required	Patients with HGG treated with anti-angiogenic agents, including bevacizumab
RANO 2.0 [17]	2D measurement with refined thresholds and minor response category	Structured qualitative assessment; NEL progression is not sufficient alone in IDH-wildtype GBM	Systematically incorporated into response interpretation	Integrated; NANO (Neurologic Assessment in Neuro-Oncology) scale discussed as an adjunct	Required mainly within the early post-radiotherapy period	Contemporary adult diffuse glioma trials across molecular subtypes and treatment modalities

HGG; high-grade glioma; RECIST; Response Evaluation Criteria in Solid Tumors; RANO; Response Assessment in Neuro-Oncology; LGG; low-grade glioma; iRANO; immunotherapy Response Assessment in Neuro-Oncology; BM; brain metastasis; NEL; non-enhancing lesion; IDH; isocitrate dehydrogenase; GBM; glioblastoma; NANO; Neurologic Assessment in Neuro-Oncology.

**Table 3:** Types of efficacy endpoints in Phase I trials. Data are summarized from our previously published analysis [36].

Endpoint	Number of Trials Including Endpoint, n/31 * (%)	Number of Efficacy Endpoints, n/94 * (%)	Commonly Used Criteria	Notes on Heterogeneity
ORR	24 (77%)	24 (26%)	RECIST, RANO, trial-specific	CNS-specific criteria are inconsistently applied
PFS	22 (71%)	22 (23%)	Investigator-defined PD	Assessment timing and PD definition varied
OS	20 (65%)	20 (21%)	—	Rarely predefined as a primary objective
DOR	9 (29%)	9 (10%)	Variable	Often post hoc
DCR	8 (26%)	8 (9%)	Variable	Definitions differed across trials

* Data are derived from a previously published analysis of 42 Phase I trials involving brain tumors. Efficacy endpoints were included as primary endpoints in 2 trials (5%) and as secondary endpoints in 31 trials (78%). The 31 trials with secondary efficacy endpoints included a total of 94 efficacy endpoints. ORR; objective response rate; RECIST; Response Evaluation Criteria in Solid Tumors; RANO; Response Assessment in Neuro-Oncology; PFS; progression-free survival; PD; progressive disease; OS; overall survival; DOR; duration of response; DCR; disease control rate.

## Data Availability

No new data were created in this study. All information is based on the previously published literature and publicly available sources.

## Abbreviations

The following abbreviations are used in this manuscript:

PFS	Progression-Free Survival
OS	Overall Survival
ORR	Objective Response Rate
DOR	Duration of Response
RECIST	Response Evaluation Criteria in Solid Tumors
RANO	Response Assessment in Neuro-Oncology
CNS	Central Nervous System
PD	Progressive Disease
PR	Partial response
DCR	Disease Control Rate
iRANO	Immunotherapy Response Assessment in Neuro-Oncology
NEL	non-enhancing lesion
2D	bidimensional
FLAIR	Fluid-Attenuated Inversion Recovery
VEGF	Vascular Endothelial Growth Factor
IDH	Isocitrate Dehydrogenase
GBM	Glioblastoma
NANO	Neurologic Assessment in Neuro-Oncology
LGG	low-grade glioma
HGG	high-grade glioma
WHO	World Health Organization
FDA	Food and Drug Administration
EMA	European Medicines Agency
PMDA	Pharmaceuticals and Medical Devices Agency
iRECIST	Immunotherapy Response Evaluation Criteria in Solid Tumors
PFS-6	6-month progression-free survival
PFS-12	12-month progression-free survival
PFS-36	36-month progression-free survival
EGFR	epidermal growth factor receptor
MGMT	O^6^-methylguanine-DNA methyltransferase
TERT	Telomerase reverse transcriptase
MRI	magnetic resonance imaging
PET	positron emission tomography
PFS2	second progression-free survival
